# Polysomnographic Indicators of Restorative Sleep and Body Mass Trajectories in the Wisconsin Sleep Cohort Study

**DOI:** 10.1093/geroni/igaa057.2178

**Published:** 2020-12-16

**Authors:** Eric Reither, Jodi Barnet, Mari Palta, Yin Liu, Erika Hagen, Paul Peppard

**Affiliations:** 1 Utah State University, Logan, Utah, United States; 2 University of Wisconsin-Madison, Madison, Wisconsin, United States

## Abstract

Previous research suggests that reductions in restorative sleep—i.e., slow-wave (N3) and rapid-eye movement (REM) sleep—are associated with weight gain and obesity in mid-to-late life. This study extends prior work by examining how within-person changes and between-person differences in restorative sleep are associated with body mass trajectories among participants in the Wisconsin Sleep Cohort Study (WSCS). We used data from 4,862 polysomnographic sleep studies and physical exams collected from 1,187 WSCS participants over an average follow-up duration of 15 years. For both men and women, we found that (1) below-average N3 and REM sleep is associated with above-average BMI, and (2) within-person loss of N3 and REM sleep is associated with larger gains in BMI, particularly between ages 30-50. Our findings highlight the importance of restorative sleep in mid-to-late life, suggesting that future clinical treatments and public health policies will benefit from heightened attention to sleep quality.

